# Effect of Daily Ingestion of Quercetin-Rich Onion Powder for 12 Weeks on Visceral Fat: A Randomised, Double-Blind, Placebo-Controlled, Parallel-Group Study

**DOI:** 10.3390/nu12010091

**Published:** 2019-12-28

**Authors:** Mie Nishimura, Takato Muro, Masuko Kobori, Jun Nishihira

**Affiliations:** 1 Department of Medical Management and Informatics, Hokkaido Information University, Nishi-Nopporo 59-2, Ebetsu, Hokkaido 069-8585, Japan; mnishimura@do-johodai.ac.jp; 2 Tohoku Agricultural Research Center, National Agriculture and Food Research Organization, Akahira 4, Shimokuriyagawa, Morioka 020-0198, Japan; muroh8@affrc.go.jp; 3 Food Research Institute, National Agriculture and Food Research Organization, Kannon-dai 2-1-12, Tsukuba, Ibaraki 305-8642, Japan; kobori@affrc.go.jp

**Keywords:** onion, quercetin, randomised controlled trial, visceral fat, alanine aminotransferase

## Abstract

Quercetin, which is frequently found in vegetables such as onion, is widely found to have biological activities such as visceral fat reduction. Therefore, we performed this randomised double-blind placebo-controlled parallel-group study and analysed the effects of daily intake of quercetin-rich onion on visceral fat for 12 weeks. Seventy healthy Japanese subjects whose body mass index (BMI) was ≥23 and <30 were recruited and randomly assigned to either the quercetin-rich onion group or placebo group. The subjects ingested 9 g of onion powder per day for 12 weeks. We conducted medical interviews, hematological and biological tests; measured body composition and vital signs; and analysed the Food Frequency Questionnaire weeks 0, 4, 8, and 12. Abdominal fat area was measured using computed tomography scanning at weeks 0 and 12. No significant differences in visceral fat area (VFA) were observed between the two groups. However, in subjects whose high-density lipoprotein cholesterol was lower, VFA was significantly lower in the quercetin-rich onion group. In addition, alanine aminotransferase was significantly lower in the quercetin-rich onion group than in the placebo group. Thus, the results suggest that quercetin-rich onion may be beneficial for preventing obesity and improving liver function.

## 1. Introduction

According to the 2016 National Health and Nutrition Survey Report of the Ministry of Health, Labour and Welfare, Japan, the proportion of the ≥20-year-old population suspected to have visceral fat obesity is 29.9% for men and 14.4% for women [1]. Visceral fat has higher fat synthesis and degradation activity than subcutaneous fat, and during fasting, visceral fat supplies free fatty acids and glycerol, which are formed on degradation of triglycerides (TGs), to the liver via the portal vein [2]. It is thought that this excessive inflow of free fatty acids and glycerol into the liver induces insulin resistance, which in turn leads to abnormal glucose metabolism, abnormal lipid metabolism and hypertension [2]. Thus, since visceral fat accumulation is related to lifestyle-related diseases, reduction of visceral fat has become an important aspect of prevention of such diseases. In 2015, the system of ‘Foods with Function Claims’ was established in Japan. Because this system permits health claims including prevention of metabolic syndrome for fresh vegetables, research on and development of various vegetables containing highly functional components is expected.

Quercetin is a flavonoid that is abundant in vegetables such as onions and in fruit, tea, and wine [3,4,5]. It exhibits antioxidant [6,7] and antihypertensive effects [8,9]. In a placebo-controlled double-blind parallel-group comparison study for subjects with body mass index (BMI) of ≥25 kg/m^2^, daily intake of tea containing 110 mg quercetin glycoside for 12 weeks significantly decreased visceral fat area (VFA) at weeks 8 and 12 after intake compared to that in the placebo food intake group [10]. The mechanism underlying suppression of fat accumulation by quercetin was indicated to involve suppression of the expression of peroxisome proliferator-activated receptor γ which is related to fat accumulation, and sterol regulatory element-binding protein 1 and fatty acid synthase which are related to fatty acid synthesis, and to increase the expression of cAMP which is related to lipolysis [11,12,13].

Onion is the one of the most popular vegetables in the world, and a major source of quercetin (28.4–48.6 mg/100 g) [14]. In the current clinical trial, we used the two quercetin-rich onion cultivars ‘Quergold’ and ‘Sarasara-gold’ to adjust the amount of quercetin contained in the test food. These onions were developed via selective breeding to develop onions containing increased amounts of quercetin. One ‘Quergold’ onion bulb (~140 g) contains 109 mg quercetin aglycone [15], and one ‘Sarasara-gold’ onion bulb (~200 g) contains 200 mg quercetin glycoside. The above findings suggest that the intake of ‘Quergold’ and ‘Sarasara-gold’ onions could provide sufficient quercetin in the range of reasonable daily volume of onion for visceral fat reduction.

Therefore, to evaluate the effects of intake of quercetin-rich onion on visceral fat, we conducted a randomised double-blind placebo-controlled parallel-group study involving healthy subjects with BMI ≥23 kg/m^2^ and <30 kg/m^2^, this range was defined as normal-high obesity and class I obesity.

## 2. Materials and Methods

### 2.1. Study Design

This study was conducted randomised, double-blind, placebo-controlled, parallel-group study over a period of 12 weeks. The data collection phase was between July and December 2018 at Hokkaido Information University, Health Information Science Center (Ebetsu, Hokkaido, Japan). Computed tomographic (CT) scans were performed by the medical doctors at the Teishinkai Central CI Clinic (Sapporo, Hokkaido, Japan). The schedule is summarized in Table 1.

Interviews were conducted by a research doctor and a nurse to obtain medical information, as well as for checking vital signs, body composition measurement and blood sampling, during the second (week 0), third (week 4), fourth (week 8) and fifth (week 12) visits. VFA, subcutaneous abdominal fat area (SFA) and total abdominal fat area (TFA) were analysed at the second and fifth visits using CT scans. In addition, the subjects filled the Food Frequency Questionnaire Based on Food Groups (FFQg; Kenpakusha, Tokyo, Japan) from the second to fifth visits.

The subjects ingested 9 g of powder (quercetin-rich onion or quercetin-free onion powder) daily for 12 weeks, at any time and in association with their favourite cooking method. Subjects daily recorded whether they ingested the test food or not, in a diary. During the entire course of this study, the subjects were asked to maintain their daily activities, including eating and exercise habits, to avoid any supplements and health foods and to restrict the consumption of onion and quercetin-rich foods. The subjects used a diary to record their daily activities, which was reviewed by a medical doctor or a nurse during each visit.

The primary outcome was the change in VFA. The secondary outcomes were TFA, SFA, body weight (BW), BMI, body fat rate (BFR), abdominal circumference, hospital blood pressure (BP), home BP and thiobarbituric acid reactive substances (TBARS). In addition, safety outcomes changed in CBCs, liver function, renal function, lipid profiles and blood glucose profiles.

### 2.2. Study Subjects

One-hundred fifty-eight volunteers were screened on their first visit; all the volunteers provided written informed consent to participate in this study. After screening, 70 healthy Japanese subjects whose BMI was ≥23 kg/m^2^ and <30 kg/m^2^ were enrolled. Inclusion and exclusion criteria are summarized in Table 2.

The methods used for assignments and blinding are briefly described: Okamoto Plant Breeding Co., Ltd., which provided the test foods, assigned the food identification numbers, which were randomly assigned to either quercetin-rich onion powder or placebo powder, and were then printed on the food packaging. Food identification number information was strictly sealed and then transferred to a third-party data center independent of the study (Media Educational Center, Hokkaido Institute of Information Technology, Ebetsu, Hokkaido, Japan). The eligible subjects were randomly assigned to the quercetin-rich onion or placebo onion groups and stratified by sex, age, and BMI during the first visit. The assignments were computer generated and based on stratified block randomisation at a third-party data center; the block size was 32 (gender (male/female), age (30s, 40s, 50s and 60s), and BMI (<24, <26, <28 and ≥28.1 kg/m^2^)). The research collaborators at Hokkaido Information University, including medical doctors, nurses, statistical analysts, and clinical research coordinators, were blinded to the food identification numbers and the assignment information during the trial period. This information was disclosed only after all the analytical data were collected and the subjects to be included in the efficacy analysis and the method to be used for statistical analyses were finalized.

### 2.3. Preparation of the Test Food

The quercetin-rich onion cultivars ‘Quergold’ and ‘Sarasara-gold’ were cultivated in Hokkaido, Japan. The quercetin-rich onion was supplied from Okamoto Plant Breeding Co., Ltd. (Hokkaido, Japan). The manufacturing process for the onion powder was as follows: peeled onions were soaked in hypochlorous acid solution for 20 min, thoroughly rinsed with water, cut into 2-mm wide pieces, dried at 45 °C for 30 h and then at 45 °C for 4 h, sterilized at 60 °C for 120 min and finally powderized. The quercetin-rich onion powder contained ‘Quergold’ and ‘Sarasara-gold’ at a ratio of 4:6. White onions, cultivated in U.S., did not have detectable levels of quercetin, and were used for the placebo powder, and the placebo powder used commercial products. The powders were analysed at Japan Food Research Laboratories (Hokkaido, Japan), the amount of quercetin was analysed at Food Research Institute, National Agriculture and Food Research Organization, and analytical results for the nutrient composition of the quercetin-rich onion and placebo powders are summarized in Table 3. Both powders were identical in appearance. 

The intake amount of quercetin in this trial was taken as a quantity for which an effect can be expected. This intake amount was decided on the basis of a previous study, which reported that 72 mg of quercetin (aglycone equivalent) leads to fat reduction [10]. Surveys in Hokkaido reported the daily intake of quercetin to be approximately 16 mg (ranging from 0.5 to 56.8 mg/day) [16]. Therefore, the daily intake was set at approximately 90 g containing approximately 60 mg of quercetin in terms of onion consumption in this trial. Since both the onion and quercetin quantities used in this trial are within the range of daily intake, it can be said that the intake in this study is a safe amount for consumption.

### 2.4. Physical, Hematological and Biological Assessments

Blood was collected from subjects after a 12-h fast and used for the following TBARS, hematological examinations: white blood cell (WBC), red blood cell (RBC), hemoglobin (Hb) and platelet (Plt) counts and hematocrit (Ht). Biological examinations were as follows: liver function (aspartate aminotransferase (AST), alanine aminotransferase (ALT), γ-glutamyltranspeptidase (γ-GTP), alanine phosphatase (ALP), and lactate dehydrogenase (LDH)); renal function (blood urea nitrogen (BUN), creatinine (CRE), and uric acid (UA)); lipid profiles (total cholesterol (TC), low-density lipoprotein cholesterol (LDL-C), high-density lipoprotein cholesterol (HDL-C) and TG); and blood glucose profiles (fasting plasma glucose (FPG), hemoglobin A1c (HbA1c) and insulin). 

The hematological tests were performed at Sapporo Clinical Laboratory, Inc. (Hokkaido, Japan). TBARS were measured using TBARS Assay Kit (Cayman Chemical, Ann Arbor, MI, USA) at Hokkaido Information University. Body composition (BW, BFR and BMI) were measured using Body Composition Analyser DC-320 (Tanita Corp, Tokyo, Japan) at Hokkaido Information University.

Hospital BP was measured by a nurse with the Automatic Blood Pressure Monitor HEM-7080IC (Omron Healthcare Co., Ltd., Kyoto, Japan), using the upper arm region of the non-dominant arm after >10-min rest. Three sequential measurements were performed, and the median of the measurements was taken at each evaluation point. Home BP was measured by the subjects with the HEM-7080IC instrument, using the upper arm region of the non-dominant arm. The subjects measured their BP daily for 1 week before visits 2–5, within 1 h after waking up (morning BP) and before going to bed (evening BP). Three sequential measurements were obtained, and the median of the measurements was taken each day. The average BP during 3 d before each evaluation point was evaluated.

### 2.5. Measurement of Abdominal Fat Area

For measurement of abdominal fat area, the subjects underwent computed tomography (CT) scanning, which was performed using a GEMINI GXL 16 system (PHILIPS, Amsterdam, Netherlands). The total fat area was calculated as the sum of the SFA and VFA. The imaging settings were as follows: tube voltage, 120 kVp; mA value, 150 mA; window level, 35; and window width, 350. A single slice on the L4 level was selected. At the time of measurement, the subjects lay on their back, with both hands up and breathing held at maximum exhalation. Abdominal body fat areas (SFA and VFA) were calculated on the basis of an abdominal CT scan image by using visceral fat measurement software (Fat Checker, J-MAC SYSTEM, INC., Sapporo, Japan).

### 2.6. FFQg

The FFQg is a semi-quantitative dietary assessment used to estimate nutrient intake on the basis of the subject’s regular diet [17]. This questionnaire included 29 food groups and 10 types of cooking methods. For each question, the subjects reported the weekly amount and frequency of food intake for the past month at each visit. From the report, regular and nutrient intakes (calorie, protein, lipid, carbohydrate, dietary fibre and sodium chloride equivalent) were estimated.

### 2.7. Safety Assessment

We considered an adverse event to be any undesirable or unintentional sign (including abnormal fluctuations in laboratory values), symptoms or illness that occurred during the food intake period. A side effect was defined as adverse events for which a causal relationship with the test food could not be completely denied (‘probably related to intake of test food’ or ‘related to intake of test food’). In this study, events that occurred between the start date of intake and the end date of intake were recorded. We further assessed the incidence of unfavourable symptoms and findings and abnormal changes in laboratory variables as adverse events. The severity of adverse events and their relationship with the test food were classified according to the protocol criteria set by the investigator. Laboratory variables related to safety outcome were assessed according to the guideline on the side effect criteria, as defined by the Japanese Society of Chemotherapy [18], and the excluded anomaly levels, as defined by the investigator. All adverse events were reported as follows: symptoms, occurrence date and severity (mild/moderate/severe); relation to test food (not relevant/probably not relevant/probably relevant/relevant/not assessable); and continuation or discontinuation, treatment, and outcome (day).

### 2.8. Ethics

This study was conducted according to the guidelines laid down in the Declaration of Helsinki (revised by the Fortaleza General Meeting of the World Medical Association), and all procedures involving human subjects were approved by the ethics committee of Hokkaido Information University (Ebetsu, Hokkaido, Japan; approved on May 25, 2018; approval number: 2018-05). Written informed consent was obtained from all subjects. This study is also in compliance with the ethical guidelines on medical research in humans as per the Ministry of Education, Culture, Sports, Science and Technology and the Ministry of Health, Labour and Welfare. This trial was registered at University Hospital Medical Information Network-Clinical Trials Registry (UMIN-CTR) (www.umin.ac.jp/ctr/index.htm; registered on 17 July 2018; registration number: UMIN000033410).

### 2.9. Statistical Analysis

Student’s *t*-tests were used to analyse the primary, secondary, FFQg and safety outcomes by comparing changes in values at baseline to those at week 4, week 8 and/or week 12 between the two subject groups. For subject characteristics, the Fisher’s exact probability test was used for sex and the Mann–Whitney *U*-test was used for the intake rate; Student’s *t*-tests were used for the other subject characteristics. All statistical analyses were performed using SPSS version 25.0 (IBM Japan, Ltd., Tokyo, Japan), and *p* < 0.05 was considered statistically significant.

### 2.10. Sample Size

The sample size was statistically determined to obtain a power of 80% with a two-sided significance level of 5%. The data from a previous clinical trial on the effect of quercetin on VFA [10] indicated that to demonstrate a difference in VFA at week 12 (postulated to have an intergroup difference of 10 cm^2^ with a standard deviation of 13 cm^2^), a sample size of 60 (30 in each group) was required. Assuming a 15% loss in the follow-up rate, 70 subjects (35 in each group) were enrolled.

## 3. Results

### 3.1. Flow Chart for Subject Involvement in Trial and Subject Characteristics

**Figure 1:**
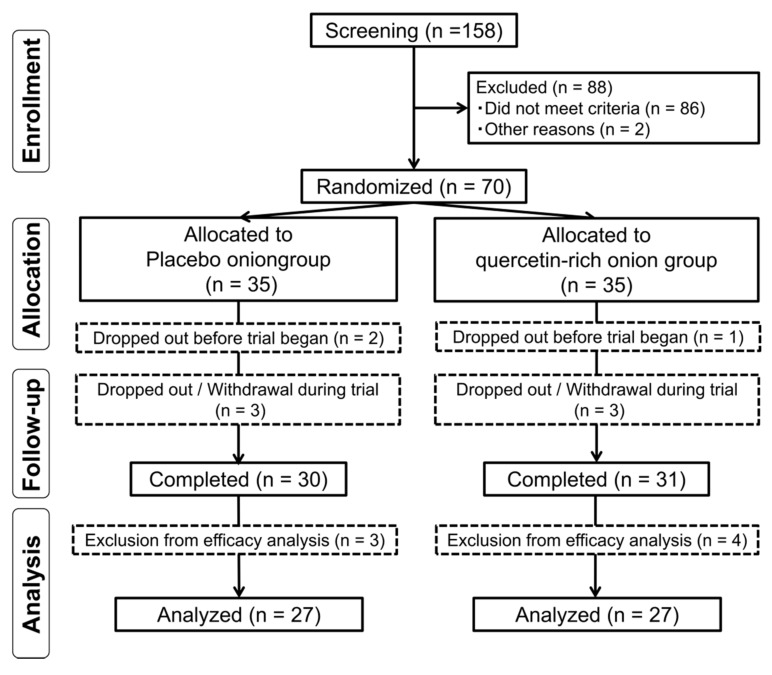
Flow chart for involvement of subjects during the trial.

A flow chart of the subject’s involvement throughout the trial schedule is presented in Figure 1. Subjects who provided informed consent (*n* = 158) were assessed for eligibility, and from these, a total of 70 subjects were enrolled in this study. All enrolled subjects were randomised to one of the two intervention groups (placebo group, *n* = 35; quercetin-rich onion group, *n* = 35). Three subjects dropped out for personal reasons before the trial started, one subject withdrew because of elevated TC at week 0 (before test food intake), two subjects withdrew because they started undergoing medical treatment, and three subjects dropped out due to personal reasons. Finally, 61 subjects completed this trial: 31 in the quercetin-rich onion group and 30 in the placebo group.

Sixty-seven subjects, excluding the three subjects who dropped out before the start of the trial, were included in the safety analysis. We excluded seven subjects from the efficacy analysis: abnormal value baseline (*n* = 2), missing data of primary outcome (*n* = 1), menopause symptoms (*n* = 1), changes in activity due to injury (*n* = 1) and diagnosis of obesity (*n* = 2). Therefore, we included 27 subjects in the quercetin-rich onion group and 27 in the placebo group. In addition, two subjects (quercetin-rich onion group) were only excluded from efficiency analysis of body composition, BP, and the oxidative marker at all points because they were absent at visit 2 (baseline), and one subject (quercetin-rich onion group) was only excluded from efficiency analysis of body composition, BP, and the oxidative marker at week 4 because of absence at visit 3 (week 4).

Data for sex ratio, mean age, body weight, BFR, BMI, abdominal circumference and intake rate for each group are showed in Table 4. These characteristics did not significantly differ between the two groups. These results proved the appropriate allocation of subjects between quercetin-rich onion and placebo groups.

### 3.2. Efficacy of Quercetin-Rich Onion in Case of VFA

The effects of quercetin-rich onion on VFA are summarized in Table 5. There were no significant differences between the quercetin-rich onion and placebo groups in terms of changes in VFA in all the subjects analysed. Next, we performed exploratory subgroup analysis for HDL-C. In subjects whose HDL-C was lower to normal (40–74 mg/dL; 74 mg/dL was the 75th percentile of baseline HDL-C-values), VFA was significantly lower in the quercetin-rich onion group than in the placebo group (*p* = 0.046).

### 3.3. Efficacy of Quercetin-Rich Onion in Case of TFA, SFA and Body Composition

The effects of quercetin-rich onion on TFA, SFA and body composition (BW, BFR, BMI and abdominal circumference) are summarized in Table 5. There were no significant differences between the quercetin-rich onion and placebo groups in terms of changes in these parameters.

### 3.4. Efficacy of Quercetin-Rich Onion in Case of BP and the Oxidative Marker

To determine the effects of quercetin-rich onion on BP, changes in hospital systolic BP (SBP), hospital diastolic BP (DBP), home SBP and DBP (morning and evening) and the oxidative marker that is TBARS, were evaluated. There were no statistically significant differences between the quercetin-rich onion and placebo groups for these parameters.

### 3.5. Assessment of Dietary Nutrients in Case of Liver Marker

As exploratory analysis, we evaluated the effects of quercetin-rich onion on liver marker, changes in AST, ALT, γ-GTP, ALP and LDH (Table 6). ALT was significantly lower in the quercetin-rich onion group than in the placebo group (*p* = 0.035).

### 3.6. Assessment of Dietary Nutrients of Subjects during the Study

For assessment of dietary nutrients, the subjects filled the FFQg (Table S1). Protein intake at week 4 and 8 (*p* = 0.008, 0.041), lipid intake at week 8 (*p* = 0.044) and dietary fibre intake at week 8 (*p* = 0.011) for the quercetin-rich onion group were significantly lower than those for the placebo group. However, there were no statistically significant differences in the intake of calories, carbohydrates, and sodium chloride equivalent between the quercetin-rich onion and placebo groups. Therefore, we consider that this difference did not affect our results. In addition, Table 3 shows that the test foods contained different amounts of sodium (placebo onion powder: 12.2 mg, quercetin-rich onion powder: 1.8 mg). However, we consider that this difference did not affect our results, as it is a minimal amount as compared to the subjects’ average daily intake of sodium, which is approximately 3,000 mg.

### 3.7. Safety Analysis

For safety analysis of quercetin-rich onion, complete blood counts (WBC count, RBC count, Hb, Ht and Plt count), renal function (BUN, CRE and UA), lipid profile (TC, LDL-C, HDL-C and TG) and blood glucose profile (FPG, HbA1c and homeostasis model assessment of insulin resistance [HOMA-IR]) were evaluated. Minimal changes (Table S2) were observed in each group. During monitoring of adverse effects (variation in clinical data and clinical observations), four subjects (placebo: *n* = 3; quercetin-rich onion group: *n* = 1) briefly experienced mild gastrointestinal symptoms after test food intake. For the other adverse events, all symptoms were mild, and the subjects recovered in a few days; the principal investigator judged that they were not related to test food intake (Table S3).

## 4. Discussion

In this clinical trial, we assessed the effect of daily quercetin-rich onion intake for 12 weeks on visceral fat. No significant differences were observed between the quercetin-rich onion and placebo groups in efficacy analysis. However, in subjects whose HDL-C was ≥40 mg/dL and <74 mg/dL, VFA was significantly lower in the quercetin-rich onion group than in the placebo group. In addition, the levels of ALT, a liver marker, were significantly lower in the quercetin-rich onion group than in the placebo group.

VFA, the primary endpoint, did not significantly differ between the quercetin-rich onion and placebo groups. However, exploratory subgroup analysis based on HDL showed that VFA was significantly improved in quercetin-rich onion group compared to the placebo group subjects with HDL-C ≥ 40 mg/dL and <74 mg/dL. A meta-analysis involving 18 studies in Asia and Australia, including 222,975 subjects, demonstrated that obesity indicators in relation to BMI and waist circumference had a stronger relationship with HDL-C and TG than with TC and/or LDL-C [19]. These findings suggested that the effect of quercetin-rich onion on visceral fat was strong in subjects with low HDL-C.

The quercetin-rich onion and placebo groups did not show significant differences with respect to the second outcome, including TFA, SFA, BW, BMI, BFR and abdominal circumference. A previous clinical trial suggested that dietary restriction for 14 d did not reduce SFA; however, VFA was found to decrease [20]. In addition, in vivo experiments in the same study showed that the expression of genes related to fat metabolism (beta3-adrenergic receptor, hormone sensitive lipase, peroxisome proliferator-activated receptor-gamma, and uncoupling protein-2) changed in the VFA because of dietary restrictions but did not change in subcutaneous fat [3]. These findings suggested that VFA was affected earlier than SFA in our trial. Moreover, the placebo group showed an increase in BFR from baseline to 12 weeks after the intake, but the quercetin-rich onion group showed suppression of this increase.

BP, including hospital BP and home BP, did not improve on intake of quercetin-rich onion. A meta-analysis study reported that an intake amount of ≥500 mg quercetin was required to obtain an effect on BP [9]. Previous clinical trial with hypertensive and obese subjects reported that BP improved on daily intake of onion epidermis extract for 6 weeks, amounting to 162 mg quercetin per day [8]. The levels of TBARS, the oxidation marker, did not improve on intake of quercetin-rich onion in our clinical trial. Quercetin exhibits antioxidant activity by regulating gene expression related to the production of reactive oxygen species [21,22]. Consumption of 1 g quercetin daily for 2 weeks significantly reduced TBARS levels after exercise in a crossover comparative study [23]. Thus, it is necessary to reconsider the intake amount of quercetin used for analysing its BP-improving and antioxidant effects.

The levels of ALT were significantly lower in the quercetin-rich onion group than in the placebo group. There are several studies on the effects of quercetin on liver function in in vivo experiments [24,25]. For example, quercetin was found to inhibit liver fat accumulation in Western-diet model mice [11]. There are two types of non-alcoholic fatty liver disease (NAFLD): simple fatty liver (non-alcoholic fatty liver [NAFL]) with mild symptoms and non-alcoholic steatohepatitis (NASH) with severe symptoms [26]; it is important to prevent progression before the onset of NASH. In this trial, although the effect of daily intake of quercetin-rich onion on VFA was limited, it can be expected to cause improvement in NAFL. In addition, VFA tended to decrease among subjects consuming the quercetin-rich onion powder whose ALT values were on the higher end of the physiological range (placebo: 1.6 ± 8.2 cm^2^, quercetin-rich onion: −6.4 ± 12.2 cm^2^, *p* = 0.060). However, this study did not include sufficient evaluation items for liver function and NAFL. Further studies are required to examine such items to verify the effect of quercetin-rich onion on liver function.

In this clinical trial, no severe side effects or severe adverse events were observed during physical and blood examinations or reported in the medical interviews. These results confirm the safety of daily intake of quercetin-rich onion for 12 weeks.

A limitation of this study is that we showed the effect of quercetin-rich onion on VFA with limited subjects. Therefore, modification of inclusion criteria, exclusion criteria and number of subjects included in the trial is required to further clarify the effects of quercetin-rich onion on VFA. In our clinical trial, ALT levels improved on consumption of quercetin-rich onion; however, the items for evaluation of liver function were limited. Therefore, we need to reconsider the variables used to prove the effect of quercetin-rich onion on liver functions. Another limitation of this study was the ingestion period; the effects of long-term ingestion need to be evaluated using other evaluation outcomes.

## 5. Conclusions

In this 12-week randomised double-blind placebo-controlled parallel-group comparative study, we assessed the effect of daily intake of quercetin-rich onion for 12 weeks on visceral fat. No significant differences were observed between the quercetin-rich onion and placebo groups in efficacy analysis. However, in the case of subjects whose HDL-C levels were low, the VFA was significantly lower in the quercetin-rich onion group. In addition, ALT was significantly lower in the quercetin-rich onion group than in the placebo group. The findings indicate that consumption of quercetin-rich onion may be beneficial for preventing obesity and improving liver function.

## Figures and Tables

**Table 1:** Clinical trial schedule.

Parameters	Guidance and Agreement	Screening	Randomisation	Wash Out Period	Test Food Intake Period			
					Week 0	Week 4	Week 8	Week 12
Visit	Visit 1				Visit 2	Visit 3	Visit 4	Visit 5
Informed consent	●							
Medical interview		●			●	●	●	●
Vital sign measurement		●			●	●	●	●
Abdominal fat measurement					●			●
Body composition measurement		●			●	●	●	●
Blood sampling		●			●	●	●	●
Home blood pressure measurement					During 1 week before each visit			
Food frequency questionnaire					●	●	●	●
Diary records				●	●	●	●	●

●: conducted.

**Table 2:** Inclusion and exclusion criteria.

Inclusion criteria	1. Age, ≥35 years and <65 years.
	2. BMI, ≥23 kg/m^2^ and <30 kg/m^2^.
Exclusion criteria	1. Subjects under physician’s advice, treatment, and/or medication for obesity and/or hypertension.
	2. Subjects with suspected obesity.
	3. Pacemaker or defibrillator users.
	4. Subjects with (or suspected to have) secondary hypertension such as renovascular hypertension, renal parenchymal hypertension, primary aldosteronism, Cushing’s syndrome, hypothyroidism and hyperthyroidism.
	5. Subjects with serious cerebrovascular, cardiac, hepatic, renal or gastrointestinal diseases and/or affected with infectious diseases requiring reports to the authorities.
	6. Subjects with major surgical history relevant to the digestive system, such as gastrectomy, gastrorhaphy and enterectomy.
	7. Subjects with unusually high and/or low BP and/or abnormal hematological data.
	8. Subjects with severe anaemia.
	9. Pre- or post-menopausal women complaining of obvious physical changes.
	10. Subjects at risk of experiencing allergic reactions to drugs or foods, especially those based on onion.
	11. Subjects regularly taking medicine, functional foods and/or supplements which would affect BW and BFR.
	12. Subjects regularly taking medicine, functional foods and/or supplements which would affect BP.
	13. Heavy smokers, alcohol addicts or subjects with disordered lifestyle.
	14. Subjects who had donated 400 mL whole blood within 16 weeks (women) or 12 weeks (men), 200 mL whole blood within 4 weeks (men and women) or blood components within 2 weeks (men and women) before the current study.
	15. Pregnant or lactating women or women who expect to be pregnant during this study.
	16. Subjects currently participating in other clinical trials or who had participated within the last 4 weeks before the current study.
	17. Any other medical and/or health reasons unfavourable to participation in the current study, as judged by the principal investigator.

BFR, body fat rate; BMI, body mass index; BW, body weight.

**Table 3:** Nutrients of test foods.

Nutrients	Placebo Onion Powder (9.0 g/day)	Quercetin-Rich Onion Powder (9.0 g/day)
Calories (kcal)	34.7	34.5
Proteins (g)	0.6	0.8
Lipids (g)	0.0	0.2
Carbohydrates (g)	7.5	7.4
Sodium chloride (mg)	12.2	1.8
Quercetin aglycone (mg)	-	60

**Table 4:** Baseline of subjects and intake rates of test foods in the quercetin-rich onion and placebo groups.

Characteristic	Placebo Onion Powder	Quercetin-Rich Onion Powder	*p*
	Mean	Mean	
Subjects (*n*)	27	27	-
Male (*n*)	2	6	0.25
Age (years)	50.4 ± 7.0	49.3 ± 8.3	0.59
Body weight (kg)	61.4 ± 6.6	63.3 ± 6.9	0.31
BFR (%)	35.3 ± 4.8	32.9 ± 5.6	0.11
BMI (kg/m^2^)	24.8 ± 1.5	24.8 ± 1.6	0.85
Abdominal circumference (cm)	85.7 ± 5.8	86.0 ± 4.5	0.84
Intake rate (%)	99.5 ± 0.9	99.4 ± 1.2	0.88

Values are shown in terms of mean and standard deviation. Fisher’s exact probability test was used for sex, Mann–Whitney *U*-test was used for intake rate and Student’s *t*-test was used for the other characteristics. *n*, number of subjects.

**Table 5:** Abdominal fat area and body composition of the placebo and quercetin-rich onion groups.

Variable		*n*	Week 0	∆Week 4	∆Week 8	∆Week 12
VFA (cm^2^)	Placebo	27	62.1 ± 27.9	-	-	−2.4 ± 10.0
	Quercetin-rich onion	27	67.5 ± 27.6	-	-	−6.0 ± 9.0
	*p*		0.47	-	-	0.16
VFA whose HDL-C is lower (cm^2^)	Placebo	18	60.9 ± 29.7	-	-	−0.7 ± 9.1
	Quercetin-rich onion	19	66.8 ± 28.8	-	-	−5.8 ± 5.8
	*p*		0.55	-	-	0.046 *
VFA whose HDL-C is higher (cm^2^)	Placebo	9	64.4 ± 25.5	-	-	−5.8 ± 11.4
	Quercetin-rich onion	8	69.4 ± 26.3	-	-	−6.6 ± 14.7
	*p*		0.70	-	-	0.91
TFA (cm^2^)	Placebo	27	282.8 ± 69.7	-	-	−8.5 ± 28.4
	Quercetin-rich onion	27	273.8 ± 59.4	-	-	−16.5 ± 29.7
	*p*		0.61	-	-	0.32
SFA (cm^2^)	Placebo	27	220.7 ± 55.4	-	-	−6.1 ± 26.3
	Quercetin-rich onion	27	206.3 ± 48.9	-	-	−10.5 ± 26.0
	*p*		0.32	-	-	0.54
BW (kg)	Placebo	27	61.1 ± 6.3	0.1 ± 0.8	0.3 ± 0.8	0.4 ± 1.1
	Quercetin-rich onion	25	62.8 ± 7.2	0.2 ± 0.7	0.2 ± 1.2	0.3 ± 1.5
	*p*		0.36	0.59	0.73	0.75
BFR (%)	Placebo	27	35.5 ± 4.9	0.5 ± 0.8	0.9 ± 0.9	1.2 ± 0.9
	Quercetin-rich onion	25	33.4 ± 5.4	0.4 ± 0.9	0.6 ± 1.1	0.5 ± 2.2
	*p*		0.15	0.65	0.30	0.14
BMI (kg/m^2^)	Placebo	27	24.7 ± 1.5	0.0 ± 0.4	0.1 ± 0.3	0.2 ± 0.5
	Quercetin-rich onion	25	24.5 ± 1.6	0.1 ± 0.3	0.1 ± 0.5	0.1 ± 0.6
	*p*		0.58	0.67	0.84	0.72
Abdominal circumference (cm)	Placebo	27	87.4 ± 6.5	−0.5 ± 2.6	−1.0 ± 3.1	−2.0 ± 3.1
	Quercetin-rich onion	25	86.9 ± 4.7	0.6 ± 2.9	−0.3 ± 2.7	−2.1 ± 3.1
	*p*		0.75	0.15	0.35	0.99

Values are shown in terms of the mean and standard deviation. Δweek 4, change in value from baseline to week 4; Δweek 8, change in value from baseline to week 8; Δweek 12, change in value from baseline to week 12. Student’s *t*-test was performed. * *p* < 0.05. VFA, visceral fat area; TFA, total abdominal fat area; SFA, subcutaneous abdominal fat area; BW, body weight; BFR, body fat rate; BMI, body mass index.

**Table 6:** Liver marker results for the placebo and quercetin-rich onion groups.

Variable		*n*	Week 0	∆Week 4	∆Week 8	∆Week 12
AST (U/L)	Placebo	27	21.1 ± 4.8	0.9 ± 4.3	1.1 ± 3.4	3.2 ± 9.2
	Quercetin-rich onion	25	22.4 ± 8.6	0.3 ± 7.0	−0.8 ± 5.8	−0.4 ± 5.9
	*p*		0.50	0.70	0.15	0.11
ALT (U/L)	Placebo	27	19.2 ± 7.9	2.4 ± 6.0	2.9 ± 6.5	6.2 ± 13.3
	Quercetin-rich onion	25	20.6 ± 9.0	3.5 ± 11.4	−0.2 ± 6.6	0.1 ± 5.2
	*p*		0.55	0.67	0.10	0.035 *
γ-GTP (U/L)	Placebo	27	21.3 ± 8.0	2.8 ± 6.3	3.7 ± 6.2	2.9 ± 7.0
	Quercetin-rich onion	25	27.7 ± 19.6	5.4 ± 12.6	5.2 ± 16.0	3.3 ± 13.6
	*p*		0.14	0.37	0.66	0.88
ALP (U/L)	Placebo	27	190.3 ± 50.1	4.6 ± 16.1	3.3 ± 19.9	4.4 ± 21.4
	Quercetin-rich onion	25	175.0 ± 36.9	11.5 ± 17.0	10.6 ± 16.5	8.2 ± 14.6
	*p*		0.22	0.14	0.16	0.46
LDH (U/L)	Placebo	27	186.4 ± 31.1	−4.4 ± 12.1	6.3 ± 19.4	−4.0 ± 15.7
	Quercetin-rich onion	25	192.4 ± 56.0	−7.7 ± 20.1	−4.9 ± 29.2	−13.1 ± 31.0
	*p*		0.63	0.48	0.11	0.19

Values are shown in terms of the mean and standard deviation. Δweek 4, change in value from baseline to week 4; Δweek 8, change in value from baseline to week 8; Δweek 12, change in value from baseline to week 12. Student’s *t*-test was performed. AST, aspartate aminotransferase; ALT, alanine aminotransferase; γ-GTP, gamma-glutamyl transpeptidase; ALP, alkaline phosphatase; LDH, lactate dehydrogenase.

## Supplementary Materials

The following are available online at https://www.mdpi.com/2072-6643/12/1/91/s1, Table S1: Dietary nutrients for the placebo and quercetin-rich onion groups, Table S2: Complete blood counts, renal function, lipid profile and blood glucose profile, Table S3: Adverse events.
