# Supplementary material for: Effect of Daily Ingestion of Quercetin-Rich Onion Powder for 12 Weeks on Visceral Fat: A Randomised, Double-Blind, Placebo-Controlled, Parallel-Group Study
Source: Nutrients. 2019 Dec 28;12(1):91. doi: 10.3390/nu12010091 (PMC7019606; doi:10.3390/nu12010091)
Supplement: Supplementary file 1 [file nutrients-12-00091-s001.pdf]

Supplementary Materials

Table S1. Dietary nutrients for the placebo and quercetin-rich onion groups

		n	Week 0	Δweek 4	Δweek 8	Δweek 12
Calorie (kcal)	Placebo	27	1737.7 ± 397.3	50.8 ± 189.3	80.5 ± 374.6	30.8 ± 300.0
	Quercetin-rich onion	27	1879.9 ± 372.3	-56.9 ± 250.2	-84.9 ± 295.8	-66.9 ± 371.2
	p		0.18	0.08	0.08	0.29
Protein (g)	Placebo	27	63.0 ± 16.1	5.2 ± 11.8	4.1 ± 14.5	3.6 ± 14.3
	Quercetin-rich onion	27	69.3 ± 16.5	-2.8 ± 9.5	-3.3 ± 11.5	0.4 ± 15.5
	p		0.16	0.008*	0.041*	0.44
Lipid (g)	Placebo	27	62.5 ± 20.1	2.7 ± 9.4	4.7 ± 18.2	1.7 ± 15.1
	Quercetin-rich onion	27	68.1 ± 17.1	-1.3 ± 11.8	-4.3 ± 13.6	-1.6 ± 14.6
	p		0.27	0.17	0.044*	0.42
Carbohydrate (g)	Placebo	27	223.7 ± 43.3	1.2 ± 25.1	5.6 ± 47.3	0.0 ± 32.4
	Quercetin-rich onion	27	234.6 ± 46.9	-8.3 ± 34.2	-7.7 ± 39.4	-13.0 ± 59.6
	p		0.38	0.25	0.27	0.32
Dietary fibre (g)	Placebo	27	13.0 ± 3.8	-0.3 ± 2.5	0.4 ± 2.4	-0.8 ± 2.9
	Quercetin-rich onion	27	13.5 ± 3.1	-1.5 ± 2.5	-1.5 ± 2.9	-1.3 ± 3.5
	p		0.64	0.08	0.011*	0.57
Sodium chloride equivalent (g)	Placebo	27	9.0 ± 2.8	0.3 ± 2.0	0.6 ± 2.7	0.2 ± 2.1
	Quercetin-rich onion	27	8.3 ± 2.2	0.2 ± 2.3	0.0 ± 2.3	0.1 ± 2.4
	p		0.29	0.88	0.40	0.93

Values are shown in terms of the mean and standard deviation. Δweek 4, change in value from baseline to week 4; Δweek 8, change in value from baseline to week 8; Δweek 12, change in value from baseline to week 12. Student's *t*-test was performed.

Table S2. Complete blood counts, renal function, lipid profile and blood glucose profile

Variable		*n*	Week 0	Δweek 4	Δweek 8	Δweek 12
WBC (×10^3/μl)	Placebo	33	5.6 ± 1.4	0.3 ± 0.9	0.1 ± 0.8	-0.1 ± 0.9
	Quercetin-rich onion	34	5.7 ± 1.4	-0.1 ± 0.7	0.0 ± 0.9	-0.1 ± 1.3
	p		0.92	0.12	0.98	0.97
RBC (×10^4/μl)	Placebo	33	459.5 ± 36.1	1.1 ± 14.2	2.3 ± 19.0	-3.0 ± 20.0
	Quercetin-rich onion	34	462.6 ± 43.9	0.9 ± 18.5	3.2 ± 19.6	0.2 ± 25.8
	p		0.76	0.96	0.96	0.60
Hb (g/dL)	Placebo	33	14.0 ± 1.0	-0.1 ± 0.5	-0.1 ± 0.6	-0.4 ± 0.6
	Quercetin-rich onion	34	13.8 ± 1.5	0.0 ± 0.5	0.0 ± 0.5	0.0 ± 0.7
	p		0.67	0.46	0.65	0.040*
Ht (%)	Placebo	33	42.3 ± 2.7	0.1 ± 1.4	1.5 ± 1.7	-0.5 ± 1.7
	Quercetin-rich onion	34	41.6 ± 3.9	0.3 ± 1.6	1.4 ± 1.8	0.1 ± 2.0
	p		0.45	0.52	0.84	0.18
Plt (×10^4/μl)	Placebo	33	27.6 ± 5.2	0.0 ± 2.6	0.2 ± 2.9	0.6 ± 2.9
	Quercetin-rich onion	34	27.1 ± 6.5	0.9 ± 2.4	1.3 ± 2.4	1.1 ± 2.5
	p		0.74	0.20	0.12	0.51

BUN (mg/dl)	Placebo	33	12.7 ± 2.7	0.5 ± 2.3	1.2 ± 2.7	1.1 ± 2.0
	Quercetin-rich onion	34	13.4 ± 3.0	1.0 ± 3.8	0.4 ± 2.4	0.8 ± 2.7
	p		0.32	0.53	0.28	0.58
CRE (mg/dl)	Placebo	33	0.8 ± 0.1	0.0 ± 0.0	0.0 ± 0.0	0.0 ± 0.1
	Quercetin-rich onion	34	0.8 ± 0.1	0.0 ± 0.0	0.0 ± 0.1	0.0 ± 0.1
	p		0.25	0.22	0.23	0.32
UA (mg/dl)	Placebo	33	4.7 ± 1.0	-0.1 ± 0.3	-0.1 ± 0.4	0.1 ± 0.5
	Quercetin-rich onion	34	5.2 ± 1.2	0.2 ± 0.6	0.1 ± 0.6	0.0 ± 0.6
	p		0.13	0.033*	0.06	0.63
TC (mg/dl)	Placebo	33	221.3 ± 33.6	5.2 ± 24.0	5.8 ± 15.9	12.0 ± 24.7
	Quercetin-rich onion	34	222.0 ± 31.4	9.5 ± 17.4	6.7 ± 17.7	1.0 ± 15.9
	p		0.93	0.45	0.83	0.049*
LDL-C (mg/dl)	Placebo	33	133.8 ± 27.2	5.1 ± 16.8	1.8 ± 13.7	8.1 ± 22.4
	Quercetin-rich onion	34	135.9 ± 29.8	8.5 ± 16.5	3.9 ± 16.7	-0.7 ± 12.3
	p		0.78	0.45	0.60	0.07
HDL-C (mg/dl)	Placebo	33	70.4 ± 14.0	0.2 ± 6.6	1.0 ± 6.2	4.0 ± 8.4
	Quercetin-rich onion	34	68.2 ± 15.0	1.6 ± 5.4	-0.7 ± 5.2	1.4 ± 6.1
	p		0.54	0.40	0.26	0.19
TG (mg/dl)	Placebo	33	97.2 ± 44.3	-3.7 ± 33.9	-12.9 ± 32.2	-11.0 ± 19.4
	Quercetin-rich onion	34	103.1 ± 47.1	5.4 ± 31.7	-0.4 ± 56.7	-11.3 ± 40.9
	p		0.61	0.30	0.31	0.97
FPG (mg/dl)	Placebo	33	88.7 ± 8.2	2.1 ± 4.1	0.5 ± 5.5	2.9 ± 4.1
	Quercetin-rich onion	34	91.0 ± 6.9	0.8 ± 3.6	-0.6 ± 5.2	2.6 ± 6.6
	p		0.23	0.23	0.44	0.81
HbA1c (%)	Placebo	33	5.4 ± 0.3	0.0 ± 0.1	0.0 ± 0.1	0.0 ± 0.1
	Quercetin-rich onion	34	5.4 ± 0.3	0.0 ± 0.1	0.0 ± 0.1	0.0 ± 0.2
	p		1.00	0.78	0.52	0.80
HOMA-IR	Placebo	33	1.3 ± 0.6	0.1 ± 0.4	0.0 ± 0.5	0.1 ± 0.5
	Quercetin-rich onion	34	1.3 ± 0.8	-0.1 ± 0.7	-0.2 ± 0.9	0.0 ± 0.9
	p		0.64	0.24	0.36	0.72

Values are shown in terms of the mean and standard deviation. Δweek 4, change in value from baseline to week 4; Δweek 8, change in value from baseline to week 8; Δweek 12, change in value from baseline to week 12. Student's t-test was performed. $p^* < 0.05$. WBC, white blood cell; RBC, red blood cell; Hb, hemoglobin; Ht, hematocrit; Plt, platelet; BUN, blood urea nitrogen; CRE, creatinine; UA, uric acid; TC, total cholesterol; LDL-C, low-density lipoprotein cholesterol; HDL-C, high-density lipoprotein cholesterol; TG, triglyceride; FPG, fasting plasma glucose; HbA1c, hemoglobin A1c; HOMA-IR, homeostasis model assessment of insulin resistance.

Table S3. Adverse events

Symptoms	Placebo, *n*	Quercetin-rich onion, *n*
Variation in liver markers	10	12
Variation in renal markers	0	2
Variation in lipid profile	4	12
Variation in CBCs	1	1
Digestive symptoms	5	1
Dizziness	1	0
Eczema	1	0
Menopausal symptoms	0	1
Throat discomfort	0	1
Dysuria	0	1
Leg injury	0	1
Swelling	0	1

CBCs, Complete blood counts.
